# One Omics Approach Does Not Rule Them All: The Metabolome and the Epigenome Join Forces in Haematological Malignancies

**DOI:** 10.3390/epigenomes5040022

**Published:** 2021-10-08

**Authors:** Antonia Kalushkova, Patrick Nylund, Alba Atienza Párraga, Andreas Lennartsson, Helena Jernberg-Wiklund

**Affiliations:** 1 Science for Life Laboratory, Department of Immunology, Genetics and Pathology, Rudbeck Laboratory, Uppsala University, 75185 Uppsala, Sweden; patrick.nylund@igp.uu.se (P.N.); alba.atienza-parraga@igp.uu.se (A.A.P.); helena.jernberg_wiklund@igp.uu.se (H.J.-W.); 2 Department of Biosciences and Nutrition, NEO, Karolinska Institutet, 14157 Huddinge, Sweden; andreas.lennartsson@ki.se

**Keywords:** epigenetic, metabolite, gene regulation, haematological malignancies

## Abstract

Aberrant DNA methylation, dysregulation of chromatin-modifying enzymes, and microRNAs (miRNAs) play a crucial role in haematological malignancies. These epimutations, with an impact on chromatin accessibility and transcriptional output, are often associated with genomic instability and the emergence of drug resistance, disease progression, and poor survival. In order to exert their functions, epigenetic enzymes utilize cellular metabolites as co-factors and are highly dependent on their availability. By affecting the expression of metabolic enzymes, epigenetic modifiers may aid the generation of metabolite signatures that could be utilized as targets and biomarkers in cancer. This interdependency remains often neglected and poorly represented in studies, despite well-established methods to study the cellular metabolome. This review critically summarizes the current knowledge in the field to provide an integral picture of the interplay between epigenomic alterations and the cellular metabolome in haematological malignancies. Our recent findings defining a distinct metabolic signature upon response to enhancer of zeste homolog 2 (EZH2) inhibition in multiple myeloma (MM) highlight how a shift of preferred metabolic pathways may potentiate novel treatments. The suggested link between the epigenome and the metabolome in haematopoietic tumours holds promise for the use of metabolic signatures as possible biomarkers of response to treatment.

## 1. Different Levels of Epigenetic Regulation with the Ability to Orchestrate Gene Expression

Methylation at the cytosine base of DNA (5mC) is the most studied epigenetic mechanism in both normal and cancerous cells. It plays numerous roles in promoter gene silencing, imprinting, X-chromosome inactivation, and genome stability, among others [1]. DNA methylation patterns are established primarily by the DNA methyltransferases 3A and 3B (DNMT3A and DNMT3B) [2], and maintained by the DNA methyltransferase 1 (DNMT1) [3]. Gene promoters contain CpG islands (CGIs), which are often unmethylated, whereas methylation of CGIs is associated with transcriptional silencing. CGIs constitute less than 10% of all CpGs in the human genome; the remainder are most often found methylated and exhibit a more diverse relationship with gene activation [4,5]. DNA demethylation occurs through passive dilution during cell division, but can also be initiated by the ten–eleven translocation (TET) family of dioxygenases through successive oxidization of 5mC to 5-hydroxymethylcytosine (5hmC), 5-formyl-cytosine (5fC), and 5-carboxylcytosine (5caC), which are excised and repaired to unmethylated cytosines by base excision repair [6,7,8,9,10]. Additionally, 5hmC itself has roles in modulating chromatin accessibility [11] and enhancer regulation [7,12,13].

In addition to DNA methylation, post-translational modifications of the histone N- and C-termini tails play crucial roles in transcriptional regulation. The most studied histone modifications include methylation, acetylation, phosphorylation, and ubiquitination, and are broadly categorised as active or repressive, based on their effects on gene expression. These histone modifications are orchestrated by the corresponding histone-modifying enzymes; methyltransferases (HMTs or lysine methyltransferases, KMTs) and acetyltransferases (HAT) deposit methyl and acetyl groups, respectively, while demethylases (HDMs or lysine demethylases, KDMs) and deacetylases (HDACs) remove them. Additionally, kinases and ubiquitin ligases deposit phosphorylation and ubiquitination, respectively, on histone proteins. Gene repression is preferentially marked by trimethylation of histone H3 lysine 27 (H3K27me3) and lysine 9 (H3K9me3), and ubiquitination of histone H2A lysine 119 (H2AK119ub) [14]. The HMT responsible for the deposition of methyl groups to H3K27 is enhancer of zeste homolog 1/2 (EZH1/2), the catalytic subunit of Polycomb repressor complex 2 (PRC2) [15], while histone H3K9 methylation is carried out by the HMT G9A (also known as euchromatic histone lysine methyltransferase 2 (EHMT2)) and is commonly associated with heterochromatin and transcriptional repression. On the opposing side, gene activation is typically supported by the trimethylation of histone H3 at lysine 4 (H3K4me3), lysine 36 (H3K36me3), and lysine 79 (H3K79me3), the acetylation or phosphorylation of histone H3 and histone H4 [16,17], and the ubiquitination of histone H2B at lysine 120 (H2BK120ub). The H3K4me3 marks are commonly found at active promoters, whereas acetylation of H3K27 is found at both promoter and enhancer regions [18]. The HMT mixed-lineage leukaemia (MLL, also known as lysine methyltransferase 2A (KMT2A)) has the ability to catalyse mono-, di-, and, to a lesser extent, trimethylation of histone H3K4 [19,20,21,22,23], and plays a pivotal role in haematological malignancies, as described below.

An additional level of epigenetic gene regulation consists of non-protein-coding RNAs, of which the most well-studied are the microRNAs (miRNAs). MicroRNAs are a class of small RNAs (15–25 nucleotides in length) with a “seed” sequence complementary to the untranslated 3′ UTR region of the target mRNA. MicroRNA binding to its cognate target most commonly leads to transcript degradation and/or transcriptional suppression, thus negatively regulating gene expression at the mRNA level. Transcription of miRNAs originates at various genomic locations, i.e., intergenic, exonic, or intronic. Upon transcription by RNA polymerase II, the pri-miRNA’s lower stem-loop structure is bound by the RNA-binding protein DGCR8 and cleaved away by the ribonuclease III enzyme DROSHA, releasing a small hairpin structure of the newly formed pre-miRNA. The pre-miRNA is then exported to the cytoplasm, where another ribonuclease III enzyme DICER, recognizes the two-nucleotide overhang generated by DROSHA and cleaves the pre-miRNA near the terminal loop, generating a small miRNA–miRNA duplex. This duplex is then loaded onto the RNA-induced silencing complex (RISC), which removes the passenger strand to generate a mature miRNA that is specific to the targeted mRNA [24,25].

## 2. Epigenetic Alterations in Haematopoietic Tumours

### 2.1. DNA Methylation Dysregulation in Haematological Malignancies

Aberrations in the DNA methylome are well-described in haematological malignancies. A large heterogeneity has been described for several types of leukaemia, both within and between patient samples [26,27,28,29]. The DNA methylation variability increases with disease progression and has a clinical and functional impact on acute myeloid leukaemia (AML), diffuse large B-cell lymphoma (DLBCL), follicular lymphoma (FL), and chronic lymphocytic leukaemia (CLL) [26,27,28,29]. Much of this variability is currently considered to arise stochastically; however, Pan et al. have recently provided evidence for the promoter methylation of DUSP22, RPRM, and SASH1 to drive disease progression and relapse in CLL [30]. Furthermore, promoter-specific DNA hypermethylation has been associated with chemotherapy resistance in DLBCL [31]. The disturbed DNA methylome is often associated with underlying genetic alterations in enzymes involved in DNA methylation, such as DNMT3A and isocitrate dehydrogenase 1/2 (IDH1/2) [32,33,34].

*DNMT3A* mutations occur in about 20% of patients with AML and about 17% of patients with T-cell acute lymphoblastic leukaemia (T-ALL), and are associated with poor disease outcome [35,36,37,38]. *DNMT3A* mutations have also been reported in myelodysplastic syndromes (MDS), chronic myelomonocytic leukaemia (CMML), and myeloproliferative neoplasms (MPN) [38]. The most common *DNMT3A* mutations impair DNMT3A methyltransferase activity and lead to CpG-specific hypomethylation [32]. *DNMT3A* mutations are thought to arise early during the AML evolution and lead to increased numbers of preleukaemic stem cells that can later evolve into AML [39]. On the other hand, DNMT1 and DNMT3B are overexpressed in T-ALL, Burkitt’s lymphoma, and DLBCL, where the overexpression is associated with advanced clinical stages and poorer responses to chemotherapy and/or radiotherapy [40,41,42].

Mutations in DNA methylation modifiers are present in about 4% of multiple myeloma (MM) patients at diagnosis and are associated with shorter overall patient survival. These include mutations in *DNMT3A*, *TET2*, and *IDH1/2* [43]. These mutations are similarly represented in human MM cell lines [44]. Although global hypomethylation in MM is associated with disease progression [45] and poor prognosis [46], at the gene-specific level, both hypo- and hypermethylation events have been linked to the regulation of genes important for MM. Hypomethylation of promoters has been suggested to be an early event in MM pathogenesis by facilitating interleukin 6 (IL-6) production [47], and has also been linked to drug resistance [48]. In turn, DNA hypermethylation in MM has been reported at numerous gene promoters [49], for some of which it is associated with shorter overall patient survival [50] and has been shown to accumulate during the course of the disease [51,52]. Notably, MM has been shown to regain high methylation levels at B-cell specific enhancers, which normally become demethylated during plasma cell differentiation, thus suggesting that MM may retain properties of early stages of haematopoietic cell differentiation [53]. DNA methylation patterns have also lately been shown to be useful in differentiating between the different molecular subgroups in newly diagnosed MM patients and to be tightly linked to chromatin modification patterns, where hypomethylated regions overlap with enrichment of H3K27me3 [54]. Recently, new data indicate that MM presents with reduced levels of 5hmC compared to normal plasma cells, and higher levels of 5hmC in MM patients were associated with early disease stages and longer overall patient survival [55]. Notably, 5hmC is thought to regulate the activity of enhancers and superenhancers in MM [56].

The gene encoding the DNA demethylating enzyme TET2 is mutated in 10–20% of AML cases, resulting in an unfavourable disease outcome [57,58,59], as well as 10–20% of MDS/MPN patients [60,61], 40–50% of patients with CMML [59,62], and about 10% of DLBCL patients [63]. Inactivation of TET2 perturbs early and late steps of myeloid and lymphoid differentiation in mice leading to the development of malignancies in these lineages [63]. During the germinal centre (GC) exit phase of B cell maturation, loss of TET2 leads to a decrease in enhancer 5hmC and the subsequent transcriptional repression of genes that guide the GC exit. This disrupts the differentiation of B cells in the GC, leading to GC hyperplasia, impaired class-switch recombination, and lymphomagenesis. The genes silenced due to TET2 impairment overlap with those normally activated by cAMP-response element-binding protein B (CREBB)-mediated histone acetylation, and mutations in *TET2* and *CREBB* are largely mutually exclusive [64]. Restoration of TET2 activity in leukaemic stem cells blocks cell self-renewal-specific DNA methylation and promotes differentiation [65]. Almost entirely mutually exclusive with *TET2* mutations in AML are mutations in *IDH1/2*, which are associated with adverse prognosis [66,67,68]. This selection for either mutant IDH1/2 or TET2 enzymes suggests a dominant transforming effect in AML [69]. *IDH1/2* mutations confer neomorphic enzyme activity, which leads to the synthesis of (R)-2-hydroxyglutarate (2-HG) [70,71,72]. 2-HG competitively inhibits TET enzymes and histone lysine demethylases of the Jumonji family, resulting in loss of 5hmC, gain of 5mC, gain of histone methylation, and a block in hematopoietic differentiation [69,73,74,75,76]. Notably, higher methylation levels are observed in mutant *IDH* than in mutant *TET2* AML [76]. Thus, the combinatorial inhibition of IDH1 and DNMTs is suggested as a potent novel strategy for the treatment of *IDH* mutant AML [77].

### 2.2. Dysregulations of Histone Modifiers in Haematological Malignancies

Mutations in chromatin modifiers are common in haematological malignancies; for example, over half of MM patients are estimated to have mutations in chromatin modifiers [78]. The MLL H3K4 methyltransferase gene was identified as a recurrent target of chromosomal translocations of the long arm (q) of chromosome 11 at band q23 in acute leukaemias, including infant, paediatric, adult, and therapy-induced AML, ALL, and acute biphenotypic leukaemia (ABL) [79,80,81,82]. In haematological malignancies, the MLL rearrangements are mostly associated with very poor prognosis, and more than 70 translocation partners to MLL have been reported [83,84]. Of these, many are themselves components of the transcriptional machinery, e.g., ELL, ENL, AF4, AF9, AF10, and CBP, or the fusion has been reported to recruit transcriptional regulators, e.g., P-TEFb, DOT1L, and Polycomb group proteins [85,86,87,88,89,90,91]. Recently, the lysine acetyltransferase KAT7, through H3K14 and H4K12 acetylation, was found to provide a platform for recruitment of the MLL fusion proteins and be essential for the proliferation of AML cells [92]. The profound transcriptional dysregulation observed in MLL-rearranged leukaemias makes it an attractive target for innovative treatment strategies [93,94,95].

Mixed-lineage leukaemia 3 (MLL3, also known as KMT2C) and mixed-lineage leukaemia 4 (MLL4, also known as KMT2D) are involved in depositing the bulk of monomethylation on H3K4 at gene enhancers and are frequently mutated in haematological malignancies [96]. The *MLL3* mutations/deletions are found in 15% of DLBCL, MDS, and AML [97,98,99]. *MLL4* mutations are observed in non-Hodgkin lymphoma (NHL) [98,100,101,102], ALL [103,104,105], and AML [106]. Mutant MLL3 and MLL4 are both suggested to contribute to lymphomagenesis in haematological cancers via the abrogation of enhancer functions during haematopoietic differentiation [107]. Lack of MLL4 impedes B-cell differentiation and class-switch recombination in the GC by perturbing H3K4me3 at target genes, some of which are known to be tumour suppressors or involved in B-cell receptor signalling pathways [108,109]. Mutations in MLL4 in human MM cell lines are suggested to contribute to resistance to dexamethasone, but also to confer sensitivity to lenalidomide [110].

Among other alterations in methyltransferases in haematological malignancies are mutations in the PRC2 methyltransferase EZH2, which deposits H3K27me3. As a matter of fact, EZH2 is the most frequently mutated Polycomb member in blood cancers [111,112]. Loss-of-function and missense mutations in EZH2 occur in AML [113,114], MDSs, CMML, primary myelofibrosis (PMF), and T-ALL [113,115,116,117,118,119]. Inactivating mutations of *EZH2* predict a poorer overall outcome in CMML, MDS, and PMF [120,121]. Heterozygous somatic mutations of *EZH2* within the histone methyltransferase (SET) domain occur in up to 30% of FL and GCB-DLBCL [122,123,124,125]. Unlike in the above cases, these are gain-of-function alterations, providing EZH2 with increased efficiency for H3K27me3 deposition and decreased efficiency for H3K27me1 deposition [126,127,128]. Expression of the heterozygous mutant EZH2 in GC B cells in mice leads to hyperplasia and B-cell lymphoma through silencing of target genes involved in cell cycle regulation and GC exit [129,130]. However, a wild-type *Ezh2* allele is required for this malignant transformation, as the phenotype of a homozygous mutant *Ezh2* resembles EZH2 loss-of-function [130]. EZH2 plays a pivotal role in guiding plasma cell differentiation, where its catalytic activity is required for preserving the preplasmablast proliferative state, and its inhibition stimulates normal plasma cell differentiation [131]. In MM, EZH2 has been defined, by our group and others, as an oncogene [132], and its overexpression is associated with poor prognosis [133]. Interestingly, no EZH2 mutations have been identified in MM to date, despite evaluation in a large number of patient samples and cell lines [43,134]. We were the first to report that the EZH2-mediated H3K27me3 deposition marks a set of genes commonly silenced in MM patients, which is also found within a signature previously associated with poorly differentiated aggressive tumours [135]. Furthermore, the silencing of the H3K27me3 targets correlates with advanced stages of the disease and poor survival [134]. These findings have led to the establishment of PRC2-mediated gene targeting as a potential therapeutic target in MM [133,134,135,136,137,138,139,140,141,142,143,144]. Combinatorial inhibition of EZH2 and DNMTs has also been suggested as a therapeutic strategy in AML [145].

In MM, mutations are observed in the H3K36me1/2 methyltransferases nuclear-receptor-binding SET domain 1 (NSD1), multiple myeloma SET-domain-containing protein (MMSET, also known as NSD2 and WHSC1), nuclear-receptor-binding SET domain 3 (NSD3, also known as WHSC1L1) [43,146], and the H3K36me3 methyltransferase SET-domain-containing 2 (SETD2). Mutations in *NSD1* and *SETD2* are either exclusively found or significantly enriched in relapsed MM patients, suggesting a role in chemotherapeutic resistance [43,78]. In about 5% of AML cases, a chromosomal translocation fuses *NSD1* to *NUP98* to generate a NUP98–NSD1 chimera, which is sufficient to induce AML in vivo [147,148]. Furthermore, a gain-of-function point mutation in the catalytic domain of *MMSET* is found in MM [149]. Additionally, in about 15% of MM cases, MMSET is overexpressed as a consequence of the chromosomal translocation t(4;14), which is associated with an adverse prognosis [150]. MMSET overexpression leads to a genome-wide increase in H3K36me2, which generates a transcriptional profile supporting myelomagenesis [151]. This increase in H3K36me2 leads to reduced global levels of H3K27me3, as well as its focal redistribution, which is thought to support MM tumourigenesis, as MMSET overexpression results in increased sensitivity to EZH2 inhibition [152,153].

Additionally, homozygous and heterozygous inactivating mutations in the H3K27me3 demethylase ubiquitously transcribed tetratricopeptide repeat on chromosome X (UTX, also known as KDM6A) have been identified in a small proportion of AML [154,155], MM lacking the chromosomal translocation t(4;14), and T-ALL [146,156], but not lymphomas that harbour EZH2 gain-of-function mutations [100]. In mice, *Utx* knockout induces spontaneous AML through global changes in H3K27 acetylation, as well as H3K4me1, ETS, and GATA binding, among others [157]. Loss of UTX in MM is thought to contribute to the malignancy by maintaining H3K27me3 gene repression, which is amenable to reactivation by EZH2 inhibition [158]. Furthermore, members of the lysine methyltransferase 2 (KMT2) family, which are known to act within a multiprotein complex together with UTX [159,160], are mutated in about 5–7% of MM patients [43,146]. Interestingly, germline autosomal dominant mutations in the lysine-specific histone demethylase 1A (LSD1, also known as KDM1A) are the first genetic alterations identified to predispose to familial MM [161].

Elevated levels of class I HDACs, particularly HDAC1, are associated with poor prognosis in MM patients [162]. Additionally, the HDAC class III sirtuin 6 (SIRT6) is overexpressed in MM and AML samples and has been described to promote adaptive genomic stability [163,164]. Recently, extensive aberrant chromatin activation marked by H3K27 acetylation was described as another unifying event in MM and was suggested to underlie crucial signalling pathways that shape the malignant plasma cell phenotype [165]. This is supported by the fact that the use of HDAC inhibition has been shown to exhibit antimyeloma effects through varying modes of action [166], and, as such, the HDAC inhibitor panobinostat is an approved treatment of MM in combination with standard therapies [167].

### 2.3. The Roles of miRNAs in Haematological Malignancies

Aberrations of miRNA expression, biogenesis, and consequent gene silencing are associated with the entire spectrum of haematological malignancies [168,169]. Expression profiling of miRNAs in blood cancers suggests their involvement as both oncogenes and tumour suppressors [170,171,172,173]. Their role often seems to be cell-type and/or context-dependent, and, as primarily based on sequence complementarity, it would also be influenced by any potential translocations and/or sequence variations, making the field particularly difficult to summarise. The myriad of functions of miRNAs can also be exemplified by their role as biomarkers for disease, treatment response, and/or disease outcome. In CLL, for example, the expression of miRNAs has been used for the fine-tuning of disease stratification [174,175]. In MM, miRNA expression signatures have been associated with the different genetic subtypes [176,177], and also suggested as regulators of the normal and malignant plasma cells [178]. Overexpression of miR-155 is described in CLL, B-cell lymphomas, including DLBCL, and cutaneous T-cell lymphoma (CTCL) [173,179,180]. In CTCL, miR-155 is involved in tumour progression [181]. In AML, miR-155 is induced by FLT3-ITD signalling and functions through targeting the transcription factor PU.1 [182]. Conversely, miR-155 has been reported to have an antileukemic role in FLT3-wild-type AML by inducing apoptosis and myelomonocytic differentiation [183]. It has also been suggested as a marker for the risk of progression from monoclonal B-cell lymphocytosis (MBL) to CLL, as well as for poor response to treatment [184]. In DLBCL, miR-155, miR-21, and miR-221 are overexpressed, and the higher expression discriminates the ABC-type immunophenotype from the GCB-type, whereas miR-21 expression is an independent prognostic indicator in de novo DLBCL [185]. In AML and ALL, overexpression of the miR-17-92 cluster is caused by either copy number amplification or direct targeting by MLL fusions, leading to increased cell proliferation and inhibition of apoptosis, suggesting that miR-17-92 may play a role in the development of MLL-rearranged leukaemia [186]. MiR-150 has been reported to be overexpressed in CLL [173] and underexpressed in chronic myelogenous leukaemia (CML), where its decreased levels are suggested to potentiate MYB and BCR-ABL expression [187]. In acute promyelocytic leukaemia, treatment with the differentiating all-*trans*-retinoic acid results in upregulation of potential tumour suppressor miRNAs, such as miR-15a, miR-15b, miR-16-1, let-7a-3, let-7c, let-7d, miR-223, miR-342, and miR-107 [188]. Reduced expression of let-7a has also been correlated with CLL pathogenesis [170]. MiR-143 and miR-145 are underexpressed in a variety of B-cell malignancies, including B-cell lymphomas, CLL cell lines, and transformed B-cell lines, suggesting that their differential expression may be used as a biomarker for malignant B cells [189]. MiR-181a has been identified as specific to B-lymphoid cells of the bone marrow, and its ectopic expression in progenitor cells leads to an increased fraction of cells within the B-cell lineage [190]. Elevated expression of miR-181a in AML can discriminate between morphological French–American–British (FAB) phenotypes and associates with the highest number of significantly correlated genes, suggesting a wide regulatory network underlying the disease [191]. Furthermore, distinct miRNA expression can distinguish AML cases with common translocations, suggesting that these miRNAs may play a role in the development of leukaemia with the associated genetic background [192]. Reduced expression of miR-204 is observed in AML, where its overexpression leads to cell apoptosis by targeting BIRC6 [193]. Moreover, expression of miR-204 potentiates sensitivity of AML cells to arsenic trioxide [194]. Overexpression of miR-125a/b in AML and MDS also exerts antioncogenic and prodifferentiation effects by modulating the NF-κB signalling pathway [195,196,197]. In MM, we have shown that the antiproliferative effects of EZH2 inhibition are concurrent with downregulation of known MM oncogenes, such as IRF-4, XBP-1, PRDM1, and MYC, which is potentially mediated by the induced expression of miR-125a-3p and miR-320c [172].

## 3. The Cellular Metabolism in Haematological Malignancies

As cancer cells acquire a cellular advantage over noncancerous cells, metabolic adaptations are required to meet the increasing need for core metabolic intermediates and macromolecules, as well as to avoid programmed cell death [198]. While multi-omics analyses of various cancers have revealed a complex and often heterogeneous pattern of clonal mutations granting a proliferative advantage [199], metabolic adaptation is widely observed across multiple malignancies and is considered to be a hallmark of cancer [200]. Furthermore, genetic alterations, including epimutations in different layers of epigenetic regulation, have an impact on the different pathways of cancer metabolism [201,202,203]. The metabolic landscape is also, in a large part, determined by the hypoxia-inducible factor 1-alpha (HIF-1α) in the hypoxic tumour environment, MYC, and other signalling pathways, including the phosphatidylinositol 3-kinase (PI3K)–AKT, NOTCH, and the mammalian target of rapamycin (mTOR) [204,205,206,207,208,209,210,211]. Therefore, the metabolic landscape for each tumour type cannot easily be described without putting it into context with cell type, mutations, epimutations, and tumour microenvironmental factors [212].

**Figure 1:**
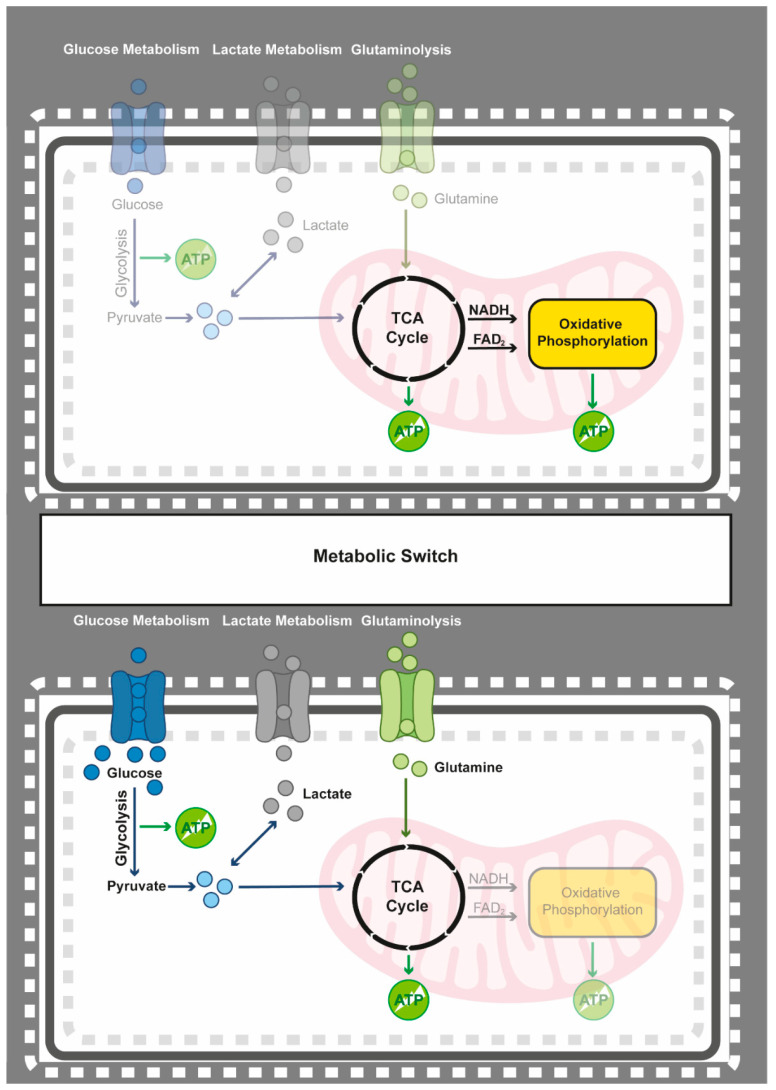
Schematic representation of the metabolic shift from oxidative phosphorylation to glycolysis. In the presence of oxygen, normal cells metabolise their glucose to pyruvate, which is then completely oxidised to CO_2_ and H_2_O via the TCA cycle and oxidative phosphorylation. Only under hypoxic conditions will normal cells reduce pyruvate to lactate through anaerobic glycolysis. Cancer cells, however, convert pyruvate into lactate, even in the presence of oxygen. In addition, while increased glucose uptake contributes to increased energy production, additional carbon sources, such as lactate and glutamine metabolism, are required to promote the synthesis of biomolecules required for rapid proliferative capacity.

Normal cellular functions dictate that energy is obtained from the generation of ATP through oxidative phosphorylation by the mitochondrial metabolism of pyruvate. However, tumour cells favour the less effective process of anaerobic glycolysis for generating ATP, which then produces lactate as a byproduct. Although the tumour microenvironment is often hypoxic, thus favouring glycolysis, cancer cells use glycolysis even when oxygen is abundant. This phenomenon, termed the Warburg effect [213], provides the cancer cells with enough reduced NADPH and glycolytic intermediates to sustain the increased need for biomolecule synthesis. In addition to the glycolytic switch, cancer cells also upregulate their glucose uptake and are prone to activate the use of alternative carbon sources (Figure 1).

**Figure 2:**
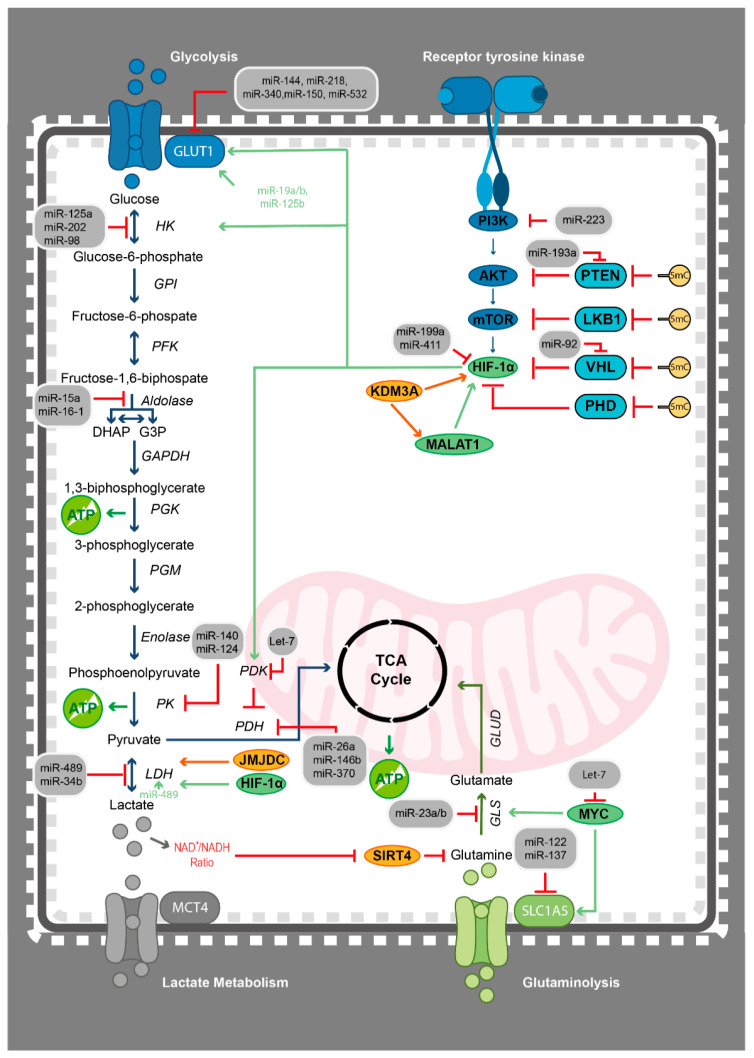
The complex regulatory network of glycolysis, lactate metabolism, and glutaminolysis is highly dependent on activation of the transcription factor HIF-1α to promote the metabolic shift from oxidative phosphorylation to glycolysis. HIF-1α participates in the glycolytic switch by regulating the expression of glucose transporters and enzymes in the glycolysis pathways. In haematological cancers, the H3K9 demethylase KDM3A contributes to an active HIF-1α phenotype and enhancement of glycolysis, while DNMT3A mediates the silencing of HIF-1α negative regulators. Additionally, miRNA regulation has a large impact on glycolysis and the access to intermediate biomolecules. MiRNAs can affect large portions of glycolysis, lactate metabolism, and glutaminolysis, which unveils a complex regulatory structure whereby miRNAs can both function as oncogenes and tumour suppressors. Red lines indicate a silencing effect. Green arrow indicates a promoting effect. All other arrows indicate enzymatic activity. GLUT1, glucose transporter type 1; HK, hexokinase; GPI, phosphoglucose isomerase; PFK, phosphofructokinase; GAPDH, G3P dehydrogenase; PGK, phosphoglycerate kinase; PGM, phosphoglyceromutase; PK, pyruvate kinase; LDH, lactate dehydrogenase; MCT4, monocarboxylate transporter 4; PDK, pyruvate dehydrogenase kinase; PDH, pyruvate dehydrogenase E1; HIF-1α, hypoxia-inducible factor 1-alpha; JMJDC, jumonji domain; SIRT4, sirtuin 4; SLC1A5, solute carrier family 1 member 5; GLS, glutaminase; GLUD, glutamate dehydrogenase; KDM3A, lysine demethylase 3A; LSD1, lysine demethylase 1A; PHD, prolyl hydroxylase domain; VHL, von Hippel-Lindau; LKB1, serine/threonine kinase 11; PTEN, phosphatase and tensin homolog; mTOR, mechanistic target of rapamycin kinase; and AKT, AKT serine/threonine kinase.

In haematological malignancies, the glycolytic switch has been well-described and is often attributed to underlying genetic alterations or essential cancer drivers. One of the most commonly altered signalling pathways in cancer is the PI3K pathway, which converges in the activation of AKT and mTOR. The PI3K/AKT/mTOR pathway has previously been associated with the regulation of pathways involving glucose uptake and glycolysis in cancer [214]. In AML, PI3K/AKT/mTOR signalling activates glycolysis [207,208]. Among others, PI3K/AKT/mTOR downstream signalling regulates the activity of HIF-1α, an important player in the metabolic shift from oxidative phosphorylation to glycolysis by regulating the expression of hexokinase 1 (HK1), hexokinase 2 (HK2), lactase dehydrogenase A (LDHA), glucose transporter type 1 (GLUT1), and pyruvate dehydrogenase kinase 1 (PDK1), all of which are vital regulators of glycolysis [215,216] (Figure 2). The activation of PDK1 results in inhibition of pyruvate dehydrogenase (PDH), thus leading to a reduced production of tricarboxylic acid (TCA) cycle substrates and, in turn, promoting glycolysis. PDKs have been shown to have important roles in disease pathogenesis in several haematological malignancies, such as CLL, AML, and CML [217,218,219]. Furthermore, MYC co-operates with HIF-1α to promote activation of the glucose transporters HK2, LDHA, and PDK1, thereby stimulating the metabolic shift from oxidative phosphorylation to glycolysis in many cancers [216,220,221]. In MLL-rearranged leukaemia, increased glycolytic activity in combination with increased levels of HIF-1α leads to resistance to inhibition of mitochondrial respiration [222], whereas inhibitors against HIF-1α are currently in development and have been proven to have antitumour activity in MM [223]. Furthermore, in MM, this metabolic reprograming may be induced by the cytokine-induced oncogenic phosphatase PRL-3 through activation of STAT1 and STAT2 transcription factors [204,205] and by cyclin D1 through targeting HK2 [206]. In MLL-AF9-induced murine AML, glycolysis is supported by AMP-activated protein kinase (AMPK) [209], whereas, in T-ALL, glycolysis is supported by the oncogenic NOTCH [210,211]. Interestingly, in B-ALL- and BCR-ABL-driven CML, inhibition of cyclin-dependent kinases (CDKs) has been shown to restrain glycolytic metabolism by a suggested mechanism involving downregulation of MYC and the rate-limiting enzymes GLUT1, HK2, and LDHA [224,225].

An additional alternative to meet the increased energy requirement of a highly proliferative cancer cell is to increase the glucose uptake. However, glucose cannot readily cross the plasma membrane of its own accord and is, thus, dependent on active transport by GLUT1, among others. GLUT1 inhibition reduces glucose uptake, resulting in increased apoptosis, and contributes to chemotherapy sensitivity in haematological cancers, such as MM and AML [226,227]. The glucose uptake in MM, has also been shown to further rely on the glucose transporter type 4 (GLUT4), targeting of which may present, as yet, an alternative treatment approach [228]. Interestingly, AML patients show signs of glucose insufficiency in the bone marrow microenvironment [229]. To overcome such glucose depletion, AML cells may utilise fructose through the upregulation of the fructose transporter GLUT5, and, as such, treatment with the fructose analogue 2,5-anhydro-d-mannitol (2,5-AM) prevents cellular proliferation in AML [229]. Alternatively, an increase in glucose uptake may be mediated by the ROS-driven upregulation of the glycolytic enzyme PFKFB3 in AML [230]. Notably, targeting glycolysis directly by using the glucose analogue 2-deoxyglucose (2-DG) has been shown to resensitise ALL cells to prednisolone treatment, albeit with severe side effects [231]. Increased glucose uptake will generate increased pyruvate that can be converted to lactate by LDHA, while generating acetyl-CoA. In fact, increased lactate production has been described in MM in combination with expression of the monocarboxylate transporter 4 (MCT4), resulting in increased lactate in the tumour microenvironment [232]. Increased lactate levels in haematological diseases could be considered as a potential marker for glycolysis-targeted intervention. Additionally, tumour cells may also activate the use of alternative carbon sources, e.g., glutamine, which can be further converted into α-ketoglutarate (α-KG) in the TCA cycle.

In contrast to the bulk of tumour cells, cancer stem cells are more dependent on oxidative phosphorylation. Indeed, metabolic analysis of leukaemia stem cells in de novo AML samples revealed that they are highly dependent on amino acid metabolism to drive oxidative phosphorylation [233,234]. Similarly, in B-ALL, a small population of stem-cell-like leukaemia-initiating cells displayed a preference in the use of oxidative phosphorylation. These cells were more resistant to cytosine arabinoside (Ara-C) treatment, an effect that could be reverted by the pharmacological inhibition of oxidative phosphorylation [235]. Similar results were shown for leukaemic stem cells in CML with minimal residual disease [236]. Furthermore, T-ALL cells have also shown dependency on oxidative phosphorylation and sensitivity towards its inhibition [237]. Additionally, CLL cells were characterised with increased oxidative phosphorylation, but not increased aerobic glycolysis [238], whereas MM cells are suggested to rely on both glycolysis and oxidative phosphorylation [239,240].

**Figure 3:**
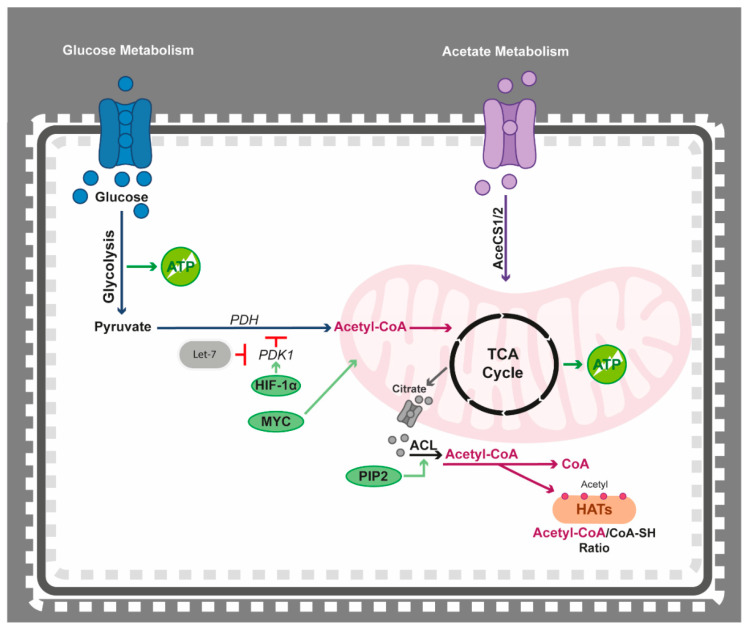
Increased glucose uptake affects the accessibility of acetyl-CoA, a key co-factor for the epigenetic regulatory machinery. Increased access to pyruvate promotes inter-mitochondrial availability, which can be used to stimulate TCA cycle activity. As mitochondrial acetyl-CoA cannot easily cross the mitochondrial membrane, active transportation of citrate from the TCA cycle is transported out of the mitochondria and converted to acetyl-CoA by ACL. High levels of acetyl-CoA promote HATs activity and can be regulated by HIF-1α by promoting activation of PDK1, which inhibits PDHs, limiting acetyl-CoA accumulation in the mitochondria. Red lines indicate inhibitory effect, while all other arrows indicate enzymatic activity. HIF-1α, hypoxia-inducible factor 1-alpha; AceCS, acetyl-CoA synthetase; PIP2, phosphatidylinositol-4,5-bisphosphate; HATs, histone acetyltransferases; PDH, pyruvate dehydrogenase E1; PDK, pyruvate dehydrogenase kinase 1; and ACL, ATP-citrate lyase.

The conversion of pyruvate during oxidation of glucose, glutaminolysis, and β-oxidation of fatty acids generates acetyl-CoA, another important molecule for the cancer cells. Acetyl-CoA is a vital molecule, as it serves as an intermediary for many metabolic pathways. Acetyl-CoA rarely moves across the mitochondrial membranes due to its polarity, so, to overcome this limitation, acetyl-CoA and oxaloacetate generate citrate in the TCA cycle, which is then translocated into the cytoplasm via the malate–citrate antiporter system (Figure 3). Citrate is then used to produce acetyl-CoA and oxaloacetate by ATP-citrate lyase (ACL) [241]. In AML, low expression of ACL has been linked to favourable prognosis [242]. Acetyl-CoA can also be synthesised from glycolysis-derived pyruvate by the action of PDH. However, active HIF-1 signalling, a common feature in haematological diseases, activates PDK and, thus, inhibits pyruvate kinase (PK) and results in less pyruvate being shuffled into the mitochondria for TCA-mediated acetyl-CoA production. In these conditions, the predominant source of acetyl-CoA is the catalysation of acetate by acetyl-CoA synthetase (AceCS) in the cytoplasm or mitochondria [243,244] (Figure 3). AceCS2 overexpression in MM has been reported to contribute to disease pathogenesis [245]. Interestingly, in a subset of MM patients, AceCS1/2 expression is increased, while levels of ACL and PDH are reduced, indicating a preferential use of acetate as a source of acetyl moieties and pointing towards a possible therapeutic vulnerability [245]. Other modes of acetyl-CoA production have been observed in haematological diseases. For instance, in glucose-deprived cells from a B-lymphoma cell line with MYC-induced activation, acetyl-CoA is solely generated through glutamine catabolism. Hence, in this context, glutamine-dependent TCA cycle function induces cellular survival and increased growth in a nutrient-poor microenvironment [246]. In addition, in AML cell lines, inhibition of glycolysis does not affect TCA cycle activity, suggesting that AML cells might utilise acetyl-CoA as an alternate energy source to glucose [247] (Figure 3).

**Figure 4:**
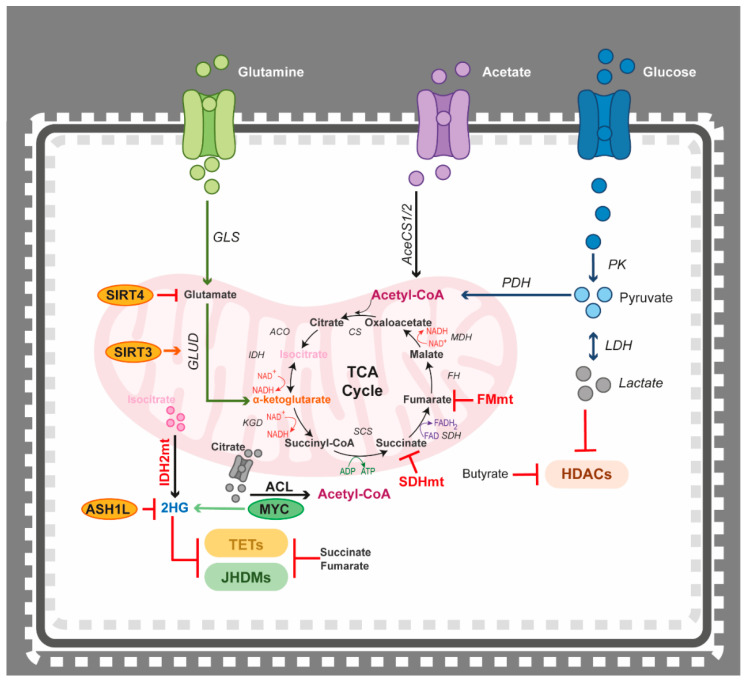
The TCA cycle is highly regulated by the access to glucose, glutamate, and acetate. Increased availability of glucose produces acetyl-CoA that can be utilised within the TCA cycle to produce citrate to be transported out of the mitochondria for acetylation conversion. In addition, increased access to glutamate allows for the TCA cycle metabolite α-ketoglutarate to accumulate, thus driving ATP production. Impairment within the TCA cycle genes has a severe impact on the epigenetic regulation. IDH2 mutation promotes accumulation of 2HG, which inhibits the TETs and JHDMs, in addition to increased levels of fumarate and succinate due to mutations in SDH or FM. Increased lactate production from pyruvate results in reduction in NAD^+^/NADH ratios, which inhibits SIRTs and HDACs function. Red lines indicate inhibitory effect and green arrows indicate a promoting effect, while all other arrows indicate enzymatic activity. GLS, glutaminase; GLUD, glutamate dehydrogenase; SIRT3, sirtuin 3; SIRT4, sirtuin 4; ASH1L, ASH1-like histone lysine methyltransferase; ACO, aconitase; IDH, isocitrate dehydrogenase; IDH2mt, isocitrate dehydrogenase 2 mutant; KGD, α-ketoglutarate dehydrogenase; SCS, succinyl-CoA synthase; SDHmt, succinate dehydrogenase mutant; SDH, succinate dehydrogenase; FH, fumarate hydratase; FMmt, fumarate hydratase mutant; CS, citrate synthase; PK, pyruvate kinase; PDH, pyruvate dehydrogenase E1; LDH, lactate dehydrogenase; HDAC, histone deacetylase; AceCS, acetyl-CoA synthetase; ACL, ATP-citrate lyase; 2HG, (R)-2-hydroxyglutarate; TET, Tet methylcytosine dioxygenase; and JHDM, lysine demethylase.

One additional metabolic pathway often dysregulated in haematological malignancies is the TCA cycle. This is caused by mutations in IDH1/2 leading to disruption of α-KG production in favour of biosynthesis of the oncometabolite 2-HG, which is associated to disease pathogenesis and progression in haematopoietic tumours. In fact, a small molecule inhibitor towards mutant IDH2, enasidenib, has been FDA-approved for refractory AML [248]. In patients with asymptomatic smouldering multiple myeloma (SMM), high levels of 2-HG are suggested to be associated with a higher risk of progression into symptomatic MM [249]. Production of α-KG can also be achieved via the increased uptake of glutamine that, in a second step, can be converted to α-KG by glutamate dehydrogenase (GLUD) (Figure 4). Depicting the importance of this metabolite, MM and ALL cell lines under depletion of glutamine have been shown to display increased sensitivity to inhibition of the antiapoptotic BCL-2. Conversely, supplementing these cell lines with an excess of α-KG limited the proapoptotic effects of conventional treatments [250,251]. Furthermore, 2-HG was recently shown to exhibit antitumour activity potentially via reducing glycolysis in wild-type *IDH* AML cells [252,253]. Additionally, disruption of the TCA cycle may be induced by the overexpression of fumarate hydratase (FH) and has been shown in AML, suggesting active fumarate/succinate catalysation [254]. Mutations in any of the succinate dehydrogenase subunits (SDHA, SDHB, SDHC, and SDHD) can cause accumulation of succinate [255]. In fact, in paediatric T-ALL and CLL, mutations in the subunit SDHB have been shown to have a potential role in preadaptation to hypoxia [256,257].

**Figure 5:**
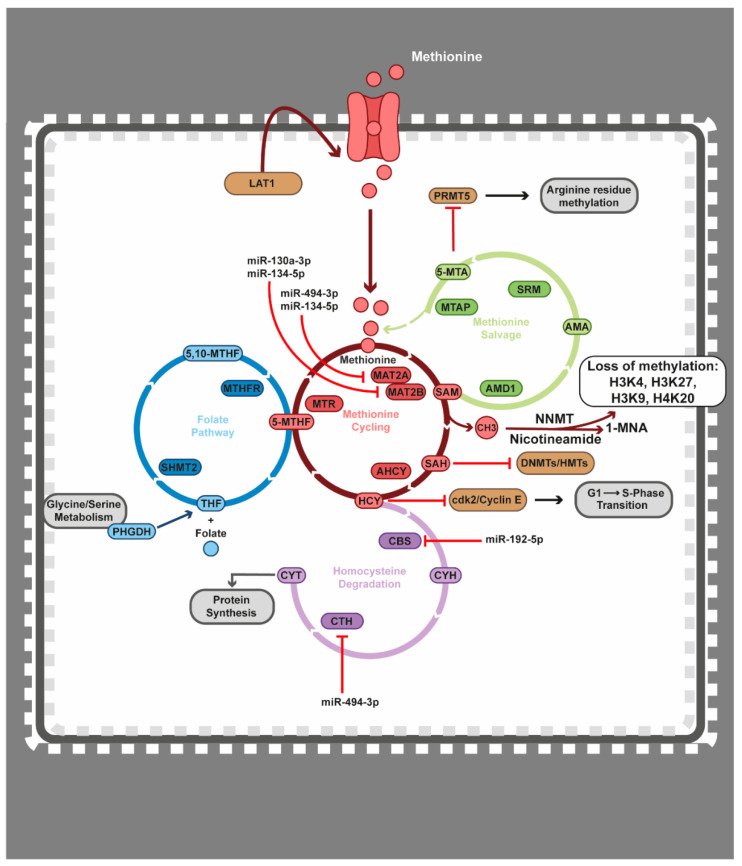
Representative illustration of the interconnection between methionine metabolism, the metabolome, and the epigenetic regulatory network. The access to methyl groups regulates the activity of histone and DNA methyltransferases. Accumulation of SAM promotes DNMTs/HMTs, while accumulation of SAH represses DNMTs/HMTs activity. A proposed miRNA-mediated silencing mechanism within methionine metabolism causes homocysteine accumulation, prevents normal cell cycle transition, and induces apoptosis in haematological malignancies. Red lines indicate inhibitory effect. 5,10-MTHF, 5, 10-methylenetetrahydrofolate; MTHFR, methylenetetrahydrofolate reductase; 5-MTHF, 5-methyltetrahydrofolic acid; MTR, 5-methyltetrahydrofolate-homocysteinemethyltransferase; THF, tetrahydrofolic acid; PHGDH, phosphoglycerate dehydrogenase; SHMT2, serinehydromethyltransferase 2; MAT2A, methionine adenosyltransferase 2A; MAT2B, methionine adenosyltransferase 2B; SAM, S-adenosylmethionine; SAH, S-adenosylhomocysteine; AHYCY, adenosylhomocysteinase; HCY, homocysteine; LAT1, L-type amino-acid transporter 1; CBS, cystathionine beta-synthase; CTH, cystathionine gamma-lyse; CYH, cystathionine; CYT, cysteine; cdk2, cycline dependent kinase 2; NNMT, nicotinamide N-methyltranferase; 1-MNA, 1-methylnicotinamide; DNMTs, DNA methyltransferases; HMTs, histone methyltransferases; AMD1, adenosylmethionine decarboxylase 1; AMA, S-adenosylmethinineamine; SRM, spermidine synthase; 5-MTA, 5′-methylthioadenosine; MTAP, methylthioadenosine phosphorylase; and PRMT5, protein arginine methyltransferase 5.

Yet another crucial cellular metabolic circuit in cancer is the methionine metabolism, which comprises of the methionine cycle, the methionine salvage, homocysteine degradation, and the folate pathways (Figure 5). Interestingly, monocytic leukaemia cells cannot proliferate in a methionine-deprived environment, despite having access to high levels of homocysteine, which is a feature of these tumour cells in large contrast to normal cells [258]. Targeting the methionine metabolism directly in MLL-rearranged leukaemias via methionine deprivation leads to induction of apoptosis, further proving the importance of this metabolite in haematopoietic tumours [259]. In MM, as well as other haematological cancers, overexpression of the L-type amino acid transporter 1 (LAT1) [260,261] results in the increased uptake of methionine [262]. In other haematopoietic tumours, e.g., T-ALL, inhibiting LAT1 has provided insight into the need for haematological cancer cells to reprogramme the cellular metabolism in order to meet their increased nutrient requirements [261]. Overexpression of 3-phosphoglycerate dehydrogenase (PHGDH) is an alternate route to meet the increased need for upregulated methionine (Figure 5). PHGDH is a rate-limiting enzyme in the glycine–serine synthesis pathway and utilizes glycolysis intermediates as donors for remethylation of tetrahydrofolate within the folate cycle. Tetrahydrofolate remethylation within the folate pathway contributes to the recycling of homocysteine to methionine [263]. Combinatorial treatment utilising a PHGDH inhibitor in MM has been shown to resensitise cancer cells to bortezomib [264]. In addition, depleting serine in leukaemic cells in combination with PHGDH inhibition resulted in reduced cell growth [265]. In Burkitt’s lymphoma, PHGDH and PSAT1 are upregulated by the action of MYC/ATF4, therefore, increasing the activity of the glycine–serine pathway [266]. Furthermore, inhibition of the folate metabolism, with molecules called antifolates, is a common therapeutic strategy in a wide range of haematological malignancies [267].

All of the above-described cellular metabolic pathways, further to playing crucial roles, are also tightly associated to epigenetic gene regulation in haematological cancers.

## 4. The Teamwork between Epigenetics and Metabolism in Haematological Malignancies

Metabolic reprogramming is key for the maintenance of viability, not only by altering nutrient influx and funnelling these nutrients into biosynthetic pathways, but also by controlling gene activity. In fact, metabolism and epigenetics are interdependent mechanisms, whereby epigenetic enzymes, on one hand, utilise metabolites as cofactors, and, in turn, have the capacity to regulate the expression of metabolic enzymes [241,268]. Several pathways are vital for global epigenetic reprogramming, such as glucose transportation, glycolysis, TCA cycle, and glutaminolysis, as well as the metabolism of lactate, methionine, lipids, and amino acids [269,270]. This interplay certainly provides a rationale for studies assessing the link between, e.g., sensitivity to epigenetic therapies and different metabolic pathways. Moreover, given the profoundly altered epigenetic system of haematopoietic tumours, as described above, examining the accompanying changes in the metabolome of these tumours has recently spurred large attention.

### 4.1. Epigenetics Teams up with the Metabolic Switch from Oxidative Phosphorylation to Glycolysis

Management of the glycolytic switch by different layers of epigenetic regulation has been observed in many haematological cancers [231,249,271,272,273,274,275]. In the first step of glycolysis, glucose is converted by hexokinase (HK) to glucose-6-phosphate, while pyruvate kinase isoform M2 (PKM2) catalyses the final dephosphorylation of phosphoenolpyruvate to pyruvate (Figure 2). Interestingly, upregulation of HK2 has been suggested to contribute to drug resistance in various haematological malignancies [276,277,278]. In paediatric AML and CML, HK2 has been shown to be directly targeted by miR-125a and miR-202, in this way disrupting glycolysis and, thus, overcoming chemoresistance [201,279]. Other miRNAs have also been demonstrated to regulate HK2, e.g., high expression of miR-98 has been associated with a good prognosis in AML patients [280,281]. In addition, in immunodeficient mice models of MM, *Hk2* knockout triggers resensitisation to proteasome inhibition, whereas PKM2 has been described to block myeloid cell differentiation [282]. Furthermore, studies show that inactivation of HK2 and PKM2 induces apoptosis and inhibition of glycolysis in myeloid leukaemic cells, as well as in MM [276,277,278]. In MM, histone deacetylase inhibitors have been shown to inhibit glucose utilization through downregulation of GLUT1 and inhibition of HK enzymatic activity [283]. In paediatric anaplastic large-cell lymphoma, the constitutive activation of the anaplastic lymphoma kinase (ALK) due to the recurrent t(2;5) (p23;q35) translocation phosphorylates PKM2, thus promoting the switch to glycolysis [284]. In CML, miR-140-5p acts as a tumour suppressor by targeting SIX1, a positive regulator of PKM2 [285]. Prior studies have demonstrated that miR-124 targets the PKM2 splicing proteins, thereby inhibiting the glycolytic metabolic rate (Figure 2) [286]. MiR-124 displays tumour-specific hypermethylation, rendering its expression silenced in ALL [287] and NHL [288].

Several regulatory mechanisms operate in glucose uptake and glycolysis, one of which includes the targeting of signalling pathways by miRNAs (Figure 2). In haematological cancers, miR-223 has been associated with both blockage of PI3K signalling and the prevention of differentiation of myeloid precursors [289]. Downstream of PI3K/AKT/mTOR signalling is HIF-1α, a crucial regulator in the shift from oxidative phosphorylation to glycolysis, as described above. In MM, miR-199a-5p has been nominated as a master regulator of HIF-1α and has been shown to be downregulated as a result of dysregulated AKT pathway [202,290], whereas miR-411-3p has been shown to inhibit HIF-1α, thus leading to a reduced cell proliferation [291]. In CLL, miR-92-1 targets VHL, a known tumour suppressor regulating HIF-1α [292]. Additional molecular mechanisms underlying the HIF-1α-mediated metabolic switch to glycolysis have been suggested, including DNMT3A-mediated promoter methylation. DNMT3A may silence the expression of the PI3K suppressors PTEN [249,293,294,295], LKB1 [270], and VHL [49,296,297], as well as the HIF-1α suppressor PHD3 [298], all in favour of PI3K activation (Figure 2). In AML, underexpression of miR-193a, a DNMT3A-targeting miRNA, triggers PTEN expression and results in PI3K cascade activation [299]. Taken together, these data suggest that dysregulation of DNMT3A may be an important regulator in promoting the metabolic shift from oxidative phosphorylation to glycolysis. The lysine-specific demethylase 3A (KDM3A), which demethylates H3K9, has also been described to stimulate the upregulation of proglycolytic genes in the glycolysis pathway. In MM, under hypoxic conditions the KDM3A is overexpressed in patient samples [300], which increases the expression of long noncoding RNA MALAT1 and contributes to accumulation of HIF-1α and upregulation of glycolysis-promoting genes [203] (Figure 2).

As an alternative to meet the increased energy requirement of a highly proliferative cancer cell is to increase the glucose uptake - expression, or loss of expression of miRNAs may, in some cases, indirectly regulate the active transport of glucose by targeting GLUT1 (Figure 2). For example, the loss of miR-144 and miR-218 has been associated with increased glucose uptake and enhanced aerobic metabolism [301,302]. Interestingly, miR-144 expression has been shown to be reduced in both peripheral blood and bone marrow samples of AML patients [303]. In alignment with this, miR-144 has been demonstrated to inhibit proliferative capability in MM [304,305], and miR-218 expression has also been demonstrated to be decreased in MM compared to normal cells [306]. In addition, upregulation of miR-144 leads to limited cell migration capacity in AML/ETO^+^ leukaemic cells [304,307]. The expression of miR-340, which is also predicted to target GLUT1, is epigenetically silenced by DNA methylation in MM [286,308], and low expression of miR-340 has been previously associated with a poor outcome in AML [309]. There are several examples of other miRNAs described as important regulators of GLUT1 in haematological cancers. In CML, loss of miR-150 expression has been shown to cause drug resistance [310,311], while it is downregulated in AML and is a critical tumour suppressor in MLL-fusion-induced leukaemogenesis [312]. MiR-532-3p has been previously associated with downregulation of GLUT1, which is accompanied by reduced proliferation in lymphoma cells [313]. Interestingly, miR-125b in T-ALL indirectly reprogrammes the metabolism by repressing the NF-кβ suppressor A20, which promotes the glycolytic switch, possibly via subsequent upregulation of GLUT1 expression [314]. In CLL, the expression of miR-125b has been found to be reduced in both aggressive and indolent cases and miR-125b overexpression led to changes in glucose, glutathione, lipid, and glycerolipid metabolism [315]. MiR-19a/b has been described to regulate GLUT1 [316,317] and is associated with poor prognosis in MM [318]. In paediatric leukaemia cells and leukaemia cell lines, expression of miR-34b is often suppressed by DNA methylation, which is suggested to affect patient response to early treatments [319]. Gain-of-function studies in MM and paediatric AML have shown that expression of miR-489 and miR-34b inhibits aerobic glycolysis by reducing glucose uptake, lactate, and ATP production by directly targeting GLUT1. In addition, upregulation of both miRNAs limited cell proliferation and induced apoptosis [320,321]. Similarly, downregulation of miR-489 leads to LDHA overexpression in MM [232]. In the second step of glucose metabolism, previous studies in leukaemia have described miR-15a and miR-16-1 as targeting the aldolase A (ALDOA), which converts fructose-1,6-bisphosphate to glyceraldehyde 3-phosphate and dihydroxyacetone phosphate in the glycolysis pathway [322] (Figure 2). Connecting glucose uptake indirectly to activation of epigenetic enzymes with dual activity, in AML, LSD1 stabilises the transcription factor GATA1 on protein level and epigenetically silences the GATA1 negative regulator C/EBPα, thus promoting glucose metabolism [323]. In haematological malignancies, e.g., AML, MDS, and CML, LSD1 contributes to leukaemogenesis [324].

In contrast, as leukaemia stem cells in de novo AML samples are highly dependent on amino acid metabolism to drive oxidative phosphorylation, combining BCL-2 inhibitor venetoclax with DNMT inhibitor azacytidine in these cells was shown to reduce oxidative phosphorylation by decreasing the usage of the amino acid metabolic pathway [233,234]. However, venetoclax/azacytidine combinatorial treatment failed to achieve the same effect in refractory/relapse patients, possibly due to the activation of nicotinamide metabolism, which likely provides an alternative source of NAD^+^ for amino acid metabolism, but also fatty acid oxidation, both of which are needed to drive oxidative phosphorylation [234,325] (Figure 2).

The activation of PDK1 by HIF-1α results in inhibition of PDH, reduced production of substrates to the TCA cycle and promotion of glycolysis. As previously mentioned, PDKs have important roles in haematological malignancies [217,218,219], while regulatory miRNAs of PDK1 have been implicated in MM and AML [326,327]. For example, let-7 is a direct inhibitor of PDK1, which, in turn, inhibits glycolysis in favour of oxidative phosphorylation through PDH activity [328] (Figure 3). PDH has also been suggested to be a direct target of miRNAs, such as miR-26a, miR-146b, and miR-370 [329,330,331]. Previous studies in haematological malignancies have shown that these miRNAs possess tumour suppressive functions [332,333,334,335].

As an alternative carbon source, tumour cells may use glutamine, which can be transported into the cell by the solute carrier family 1 member 5 (SLC1A5). Studies have shown that miR-137 and miR-122 directly inhibit SLC1A5, resulting in downregulation of glutamine metabolism [336] (Figure 2). Interestingly, miR-137 has been demonstrated to be epigenetically silenced by promoter hypermethylation in MM and ALL cells [337,338]. In addition, low expression of miR-122 has been associated with poor prognosis in AML [339]. MiR-23a/b directly regulates glutaminase (GLS), the enzyme responsible for the conversion of glutamine to glutamate, needed for the generation of α-KG (Figure 2). In B-cell lymphoma, MYC-mediated suppression of miR-23a/b results in increased glutamine metabolism [340]. Interestingly, overexpression of miR-23a causes a reduction in GLS expression and promotes cell death in leukaemic cells [340]. Sirtuins (SIRTs), or HDACs class III, have also been shown to have relevant implications for the increased need for glycolysis, glutaminolysis, and lipid metabolism [341]. SIRTs are dependent on the availability of NAD^+^ and the NAD^+^/NADH ratio is highly connected to energy output (Figure 2). Lactate conversion to pyruvate requires the reduction in NAD^+^ to NADH, while the reverse reaction requires NADH, which results in NAD^+^ increase. As such, the NAD^+^/NADH ratio is closely related to the availability of pyruvate and lactate, as well as the enzymatic activity of LDHA [342]. When cellular energy is high, the NAD^+^/NADH ratio drops and a genome-wide nonacetylated state is assumed [343]. SIRT6 causes deacetylation of H3K9 and histone H4 lysine 56 (H4K56), as well as non-histone proteins, and its activity has also been described to alter cellular glucose metabolism by deacetylating PGC-1α, thus promoting increased gluconeogenesis [344].

### 4.2. Epigenetics Teams up with Acetyl-CoA

Acetyl groups are essential for cellular substrate-dependent mechanisms, such as the epigenetic action of HATs, exemplifying that not only does the epigenome regulate the metabolome, but the converse is also true. Access to high levels of acetyl-CoA promotes cellular growth, lipid synthesis, and histone acetylation. In the cytoplasm, citrate is used to produce acetyl-CoA with the help of ACL (Figure 3) [241]. ACL activity correlates with increased histone acetylation in cancer cells [345,346] and ACL knockdown decreases histone acetylation and disrupts the expression of glycolysis-related genes [346]. The PI3K pathway is commonly dysregulated in cancer due to mutations in PI3K/PTEN and/or upstream activators, such as the RAS family of proteins. When activated, PI3K catalyses the conversion of phosphatidylinositol-4,5-bisphosphate (PIP2) into phosphatidylinositol-3,4,5-triphosphate (PIP3), the messenger molecule that signals activation to downstream effectors, including AKT (Figure 3). In AML, PIP2 binding to ACL enhances its activity, whereas inhibitors against PI3K reduce ACL activity and acetyl-CoA availability, resulting in blockage of H3K9 acetylation [347]. Upon active HIF-1 signalling, less pyruvate is available for TCA-mediated acetyl-CoA production and the predominant source of acetyl-CoA is the catalysation of acetate by AceCS, as described above [243,244] (Figure 3). In MM, AceCS2 contributes to disease pathogenesis via increased acetylation and stabilisation of the known oncogene IRF4 [245].

In summary, due to the vital role of the deposition of acetyl groups on key residues within the chromatin landscape, many of the enzymes regulating the availability of acetyl-CoA are dysregulated in haematological malignancies to maintain a favourable tumour transcriptional environment.

### 4.3. Epigenetics Teams up with the Tricarboxylic Acid (TCA) Cycle

Similar to acetyl-CoA, the TCA intermediate metabolite α-KG acts as a cofactor for a number of epigenetic enzymes. These include the DNA demethylating enzymes TET1/2, as well as the members of the Jumonji-domain histone demethylase (JHDM) family. Mutations in IDH1/2 cause disruption of α-KG production and synthesis of the oncometabolite 2-HG. Linking the activity of 2-HG to epigenetic regulation, prior studies have shown that 2-HG has an inhibitory effect on the α-KG-dependent TET1/2 and JHDM2A/KDM3A enzymes [72,75] (Figure 4). As mentioned in the section above, approximately 20% of AML and T-cell lymphoma patients carry mutations in the *IDH* gene, thus resulting in disease-specific histone and DNA hypermethylation signatures [69,73,348]. Enasidenib is an FDA-approved small molecule inhibitor of mutant IDH2 that has, in experimental mouse xenograft models of AML, proven to reduce serum levels of 2-HG and decrease DNA and histone methylation [248]. Loss of the histone lysine methyltransferase ASH1L has been described to lead to upregulation of HOXA9 in AML, which co-operates with mutant IDH2 in accelerating leukaemogenesis [349]. In addition, a decrease in α-KG has been shown to cause hypermethylation in AML samples, similar to what is observed in AML patients carrying *IDH2* mutations [350].

Lysine demethylases of the JHDM family, such as the TET proteins, are dependent on α-KG from the TCA cycle as a cofactor and are inhibited by succinate and fumarate accumulation [351] (Figure 4). In cancer, alterations in both FH and succinate dehydrogenase (SDH) can lead to disruption in the α-KG/fumarate/succinate balance, causing an inhibitory effect on TET and JHDM activity and leading to aberrant DNA and histone methylation [351,352,353]. In DLBCL, impairment of the SIRT3 histone deacetylase has significant negative effects on glutaminolysis, which fuels the TCA cycle [354]. Glutamate is converted to α-KG by GLUD to release stored energy by oxidation through the TCA cycle. Deacetylation of GLUD by SIRT3 is vital for GLUD enzymatic activity, and, thus, is critical for glutamate-dependent DLBCL cells [354]. SIRT4 expression has been detected when treating primary AML cells with histone deacetylase inhibitors. However, SIRT4 has an inhibitory effect on glutamine metabolism, serving as a metabolic block by repressing glutamine uptake into the TCA cycle, which, in turn, contributes to cell cycle G1 arrest [355]. This process is made possible by the repression of mitochondrial GLUD [356] (Figure 2). Interestingly, deacetylation capacity is highly dependent on and regulated by metabolic activity and rate-limiting macromolecules. In fact, deacetylation by HDACs can be metabolically antagonized by butyrate. In a murine model of lymphoma, high intake of butyrate through extensive fibre consumption has been shown to reduce cancer cell growth in combination with dose-dependent induction of apoptosis and histone deacetylation [357]. Furthermore, accumulation of lactate has also been shown to reduce HDAC activity [358] (Figure 4).

### 4.4. Epigenetics Teams up with the Methionine Cycle

DNMTs and HMTs are highly regulated by access to methyl groups in the form of S-adenosyl-methionine (SAM), which is derived from methionine metabolism (Figure 5). In the first step, the methionine adenosyltransferase 2A/B (MAT2A/MAT2B) complex converts methionine to SAM. This process generates the byproduct S-adenosyl-homocysteine (SAH), which, in a negative feedback loop, strongly inhibits DNMTs and HMTs [270]. In the second step, SAH is converted to homocysteine (HCY) by adenosyl homocysteinase (AHCY). Interestingly, introducing a competitive inhibitor to AHCY in combination with DNMT inhibition results in the reactivation of potent tumour suppressor genes in AML cells [359]. In the third step, HCY can either be degraded by cystathionine gamma-lyase (CTH) and cystathionine beta synthase (CBS) enzymes, or become remethylated by the donation of a methyl group from 5-methyltetrahydrofolic acid (5-MTHF), which is catalysed by the 5-methylhydrofolate-homocysteine methyltransferase (MTR) enzyme in the folate pathway [360,361] (Figure 5). Increased cellular access to SAM alone has been demonstrated to cause DNA hypermethylation [362]. In MLL-rearranged leukaemia methionine deprivation leads to an overall loss of cellular methylation potential and induction of apoptosis [259]. As mentioned above, overexpression of PHGDH is an alternate route to meet the increased need for upregulated methylation. In Burkitt’s lymphoma, chemical inhibition of PHGDH resulted in decreased DNA and histone methylation, reactivation of tumour suppressor genes, and decreased proliferation [266].

SAM can also be consumed during the conversion of nicotinamide to 1-methylnicotinamide (1-MNA) by nicotinamide N-methyltransferase (NNMT) (Figure 5). As a master regulator of SAM accessibility, overexpression of NNMT causes rapid SAM depletion and limits the availability of methyl donors to DNMTs and HMTs. In addition, NNMT overexpression contributes to maintaining the pluripotency of embryonic stem cells [363]. Cells overexpressing NNMT show a complete depletion of H3K4, H3K9, H3K27, and H4K20 methylation, resulting in dysregulation of key signalling pathways and allowing for an undifferentiated pluripotent phenotype [364]. In addition, polymorphisms in NNMT have been associated with a prognostic risk of developing paediatric ALL [365].

The link between methionine metabolism and drug response was recently demonstrated by us in MM [366]. In this study, we utilised cellular metabolic profiling to determine the response to an epigenetic-targeted intervention. This metabolomics profiling revealed that inhibition of the HMT EZH2 impaired the methionine metabolism pathways by downregulation of *MAT2A*, *MAT2B*, *CBS*, and *CTH*. This effect was mediated by the reactivation of miR-494-3p, miR-130a-3p, miR-134-5p, and miR-192-5p. Interestingly, these miRNAs have previously been connected with drug sensitivity, response, and prognosis in other haematological cancers [367,368,369,370,371]. The microRNA-mediated gene downregulation was accompanied by the accumulation of HCY and 5-MTHF in the methionine cycling pathway, and 5-methylthioadenosine (5-MTA) in the methionine salvage pathway. 5-MTA acts as a competitive inhibitor of the arginine methyltransferase PRMT5 (Figure 5), which has been shown to promote cell cycle progression in combination with PI3K-AKT activation in DLBCL [372]. Furthermore, methylthioadenosine phosphorylase (MTAP) loss in T-ALL has been demonstrated as a therapeutic vulnerability due to limiting PRMT5 activity [373] (Figure 5). These results suggest that metabolic profiling should be considered a significant and relevant tool to determine cell sensitivity to epigenetic treatments.

## 5. Conclusions and Future Directions

Genetic and gene expression profiling are readily used to study cancer biology, disease progression, and response to treatment. Whole-genome epigenomic studies are closely catching up to also being widely used, as epigenetic gene regulation by DNA methylation, miRNA-associated gene repression, and histone modifications is now being recognised as equally important in the onset and progression of the disease. Needless to say, the tightly intertwined collaboration between the cellular genetic background, the epigenetic machineries in play, and the gene expression output are becoming a central part in understanding disease. However, the mechanisms by which the metabolism regulates gene expression are often neglected and ill-appreciated, although the methods to study whole-cell metabolites, such as liquid chromatography–mass spectrometry, have existed for decades. Furthermore, although an integrated understanding of the epigenetic and metabolic interplay in cancer is far from complete, it is well-established that the cellular metabolic status both influences and is influenced by the epigenome. Firstly, the accessibility to co-factors needed by epigenetic enzymes is commonly altered due to reprogramming of the cellular metabolism. Hence, metabolic rewiring in cancer has the potential to hijack the epigenetic machinery in order to produce a gene expression profile that favours increased proliferation. Secondly, epigenetic changes regulate the expression of key enzymes in metabolic pathways, thereby modulating the access to key biomolecules. This interdependency may disclose previously unrecognised therapeutic vulnerabilities that, alone or in combinatorial regimens, could open for yet novel therapeutic approaches for combatting cancer.

Dysregulation of the epigenetic machinery is well-documented in haematological cancers, in some cases, also as a driver event, e.g., mutated IDH1/2 in AML. Two main characteristics of epigenetic regulation make it an exceptional target for therapeutic intervention. On one hand, unlike genetic defects, all epigenetic mechanisms are reversible and, therefore, amenable for treatment. On the other hand, epigenetics acts at the genome scale by regulating multiple loci at the same time, and thus the targeting of individual epigenetic enzymes has an effect on a multitude of genes, reducing the likelihood of developing resistance. Reflecting this, epigenetic treatments are now part of the standard of care for several haematological malignancies; DNMT inhibitors are currently used for the treatment of AML and MDS, and HDACs are FDA-approved for the treatment of MM, CTCL, and peripheral T-cell lymphoma [374]. Additionally, at the time that this review was written, there were 83 registered clinical studies for epigenetic inhibitors in cancer (https://clinicaltrials.gov/; accessed on 6 July 2021). Likewise, several drugs directed towards the abnormal cancer metabolism are currently used in clinical practice. In fact, two of the first FDA-approved pharmacological treatments for cancer, i.e., leucovorin and methotrexate, interfere with folate metabolism [375], and more metabolism-targeting drugs are at different phases of clinical validation [376].

To fully utilise the potential of the interplay between metabolism and epigenetic modulators, future clinical trials in these and other areas should incorporate the analysis of biomarkers to unravel not only alterations to the transcriptome, but also to the metabolome, thus allowing for the stratification of patients that might benefit from targeted treatments and providing a tool for the monitoring of the response over time. This is facilitated by the fact that metabolites can be readily detected by using noninvasive methods in, for instance, plasma. Exemplifying this, the recent study from our research group provides new insights into the metabolic response to targeted epigenetic treatment in MM and suggests that metabolic profiles have potential as biomarkers for the response to EZH2 inhibition in MM. Furthermore, given the extensive association between epigenetics and metabolism, combination approaches involving dual inhibition of epigenetic and metabolic regulators may certainly hold promise for tumour growth inhibition.
